# Anoctamin-1 affects the migration and invasion of anaplastic thyroid carcinoma cells

**DOI:** 10.1080/19768354.2019.1614981

**Published:** 2019-05-10

**Authors:** Jae-Young Kim, Hwa Young Youn, June Choi, Seung Kuk Baek, Soon Young Kwon, Bok Kee Eun, Jae-Yong Park, Kyoung Ho Oh

**Affiliations:** a Department of Veterinary Internal Medicine, College of Veterinary Medicine, Seoul National University, Seoul, Republic of Korea; b Department of Otorhinolaryngology-Head and Neck Surgery, Korea University College of Medicine, Seoul, Republic of Korea; c Core-Laboratory for Convergent Translational Research, Korea University College of Medicine, Seoul, Republic of Korea; d School of Biosystem and Biomedical Science, College of Health Science, Korea University, Seoul, Republic of Korea

**Keywords:** Anoctamin, thyroid cancer, invasion, migration

## Abstract

Anaplastic thyroid carcinoma (ATC) is a rare malignancy with very poor prognosis. The exact cause underlying its strong aggressive nature is not clear. Here, we discovered the elevated expression of anoctamin-1 (ANO1; Ca^2+^-activated Cl^−^ channels) in advanced-stage ATC tissue. Using different ATC cell lines, the degree of expression of ANO1 was found to be related to the degree of ATC cell invasion by quantitative reverse transcription polymerase chain reaction and western blotting. Suppression of ANO1 activity either by selective inhibitor (T16Ainh-A01) or by siRNA significantly attenuated the migration and invasion of ATC cells. In conclusion, ANO1 appears to increase the ability of ATC cells to invade and migrate. Our results also suggest that the expression of ANO1 in patients with ATC may be helpful in predicting the prognosis of ATC.

## 1. Introduction

Anaplastic thyroid carcinoma (ATC) is a rare malignancy of the thyroid gland (Molinaro et al. 2017). Only 1–2% of all thyroid cancers are anaplastic, although anaplastic disease accounts for 14–50% of the annual mortality from thyroid cancer, with a median survival time of 3–5 months (Demeter et al. 1991; Kebebew et al. 2005). Invasion and metastasis are the leading causes of cancer fatality. Invasion of ATC into the trachea, larynx, esophagus, recurrent nerve, or common carotid artery is common (McIver et al. 2001). Hence, surgical resection based on clear histological diagnosis is the preferred treatment. The rare nature of this disease poses a challenge by making protocol allocation difficult. At present, no effective therapy for ATC exists and no proper treatment is available for patients with ATC. Efforts have been directed to better understand the molecular pathogenesis and clinical course of ATC (O'Neill and Shaha 2013). Recent advances in the understanding of the molecular and genetic pathogenesis of ATC may allow the development of targeted therapy for this disease (Kebebew et al. 2005; Nagaiah et al. 2011).

Voltage-gated ion channels are known to play a significant role in several types of cancers (Diss et al. 2005; Rao et al. 2015). Understanding the role of ion channels in cancer is a novel area in oncology research, and it has been reported that ion channels are related to proliferation, invasion, migration, and metastasis of different cancer types (Prevarskaya et al. 2011). Ca^2+^-activated Cl^−^ channels (CaCCs) have critical functions in physiological processes, including epithelial secretion, olfactory perception, control of neuronal and cardiac excitability, regulation of smooth muscle contraction, and nociception (Perez-Cornejo et al. 2012). Anoctamin-1 (ANO1) was identified as a CaCC (Caputo et al. 2008; Yang et al. 2008). ANO1 is found on the cell plasma membranes in various tissues, including cardiovascular endothelia and gastrointestinal epithelia, and is thought to contribute to maintaining homeostatic chloride flux (Hartzell et al. 2005). ANO1 mediates Ca^2+^-dependent Cl^−^ fluid secretion, cell volume regulation (Huang et al. 2012; Catalan et al. 2015), and supports the contraction of non-neuronal non-muscular Cajal cells and smooth muscle cells in the epididymis and oviduct (Hwang et al. 2009; Zhu et al. 2009).

Some reports showed that elevated expression of ANO1 in head and neck squamous cell carcinoma, breast cancer, lung cancer, and prostate carcinoma leads to increased tumor growth and cell invasion (Liu et al. 2012; Ruiz et al. 2012; Jia et al. 2015). The correlation between ANO1 and tumorigenesis has gained significant attention following a recent study evaluating ANO1 expression in different types of cancers (Duvvuri et al. 2012). However, the relationship between thyroid cancer and ANO1 has not yet been reported. To address this question, we analyzed the expression of ANO1 in human ATC tissues, and identified the biological function of ANO1 in the malignancy of ATC cell lines. Our results suggest that increased expression of *ANO1* may promote migration and invasion properties of malignant ATC cells.

## 2. Materials and methods

### 2.1. Human tissues

Human tissues were obtained from the Department of Otolaryngology-Head and Neck Surgery, Korea University Ansan Hospital. In all cases, the protocols of the study were approved by the Institutional Review Board committee. A total of 44 thyroid tumors, 4 anaplastic thyroid carcinomas, 20 papillary thyroid carcinomas, and 20 thyroid hyperplastic nodules were analyzed.

### 2.2. Cell culture

SNU-80 and KTC cells were obtained from the Korean Cell Line Bank (KCLB, Seoul, Korea), and FRO cells were kindly provided by Professor Seung Kuk Baek (Korea University, Korea); all three cell lines are ATC cell lines. Cells were cultured in Roswell Park Memorial Institute (RPMI)-1640 medium supplemented with 10% fetal bovine serum (FBS) and l-glutamine (300 mg/L). All cell lines were grown in plastic culture flasks (VWR, Canada) incubated at 37°C in a humidified atmosphere containing 5% CO_2_. Cells were subcultured at 72 h-intervals using 0.25% trypsin-0.02% ethylenediaminetetraacetic acid (EDTA) and seeded into fresh medium at a density of 2.5–3.5 × 10^5^ cells/mL.

### 2.3. Immunofluorescence microscopy

Immunofluorescence staining of tissues was performed according to the standard protocol. Briefly, paraffin-embedded tissue samples were rehydrated and antigen retrieval was applied to unmask epitopes altered by 10% formaldehyde fixation. Sections were incubated overnight with the primary anti-TMEM16A antibody (1:500; ab53212, Abcam, Cambridge, UK) in a blocking buffer. The slides were washed three times for 10 min each with phosphate-buffered saline and incubated with the secondary antibody (1:2000; Alexa Fluor 488 donkey anti-rabbit IgG, Molecular Probes by Life Technologies, USA) and a nucleic acid stain solution (1:5000, Hoechst 33342 trihydrochloride, Life Technologies Corporation, USA). The samples were washed three times for 10 min each with wash buffer and mounted with an anti-fade mounting medium. The slides were observed under a confocal laser microscope.

### 2.4. Western blot analysis

Total cells were lysed with radioimmunoprecipitation assay (RIPA) buffer (0.22% beta-glycerophosphate, 10% tergitol-NP40, 0.18% sodium orthovanadate, 5% sodium deoxycholate, 0.38% ethylene glycol tetraacetic acid (EGTA), 1% sodium dodecyl sulfate (SDS), 6.1% Tris, 0.29% EDTA, 8.8% sodium chloride, and 1.12% sodium pyrophosphate decahydrate) containing protease inhibitor cocktail tablets (Roche, Germany). Protein concentration was determined with a bicinchoninic acid (BCA) protein assay kit (Fisher Scientific, Waltham, MA, USA). Equal amounts of protein lysates (20 μg) were separated with SDS-polyacrylamide gel electrophoresis (PAGE) (Invitrogen, USA) and the proteins were electrophoretically transferred onto nitrocellulose membranes. The blotting membrane was blocked with 5% milk in Tris-buffered saline containing 0.1% Tween-20 (TBS-T buffer) and incubated overnight at 4°C with rabbit anti-TMEM16A (1:1000) and mouse anti-glyceraldehyde-3-phosphate (GAPDH) (1:1000; sc-32233, Santa Cruz, CA, USA). The membranes were washed with TBS and incubated with anti-rabbit or -mouse lgG-horseradish peroxidase (HRP) secondary antibody (1:5000, Jackson Lab, USA) for 1 h. For protein visualization, the nitrocellulose membranes were incubated with western blot detection reagents (enhanced chemiluminescence, West Dura, and Femto) prior to their exposure on KODAK film.

### 2.5. Transfection with siRNA

For transfection with siRNA targeted toward *ANO1*, three siRNAs were introduced into the FRO cell line using Lipofectamine 2000 (Invitrogen, Carlsbad, CA, USA) according to the manufacturer’s protocol. The hTMEM16A siRNAs (BIONEER, Korea) used in this study are described as follows: siRNA-1 (1152920) forward, GUC CAA CAU CCG GGU CAC and reverse UGU GAC CCG GAU GUU GGA; siRNA-2 (1152922) forward, CUG UUU GCG CUG CUG AAC A and reverse UGU UCA GCA GCG CAA ACA G; siRNA-3 (1152925) forward, UGA AUC CUC CUA AUU CCU U and reverse AAG GAA UUA GGA GGA UUC A.

### 2.6. Quantitative real-time reverse transcription polymerase chain reaction (qRT-PCR)

RNA was isolated using the RNeasy Plus Mini kit (QIAGEN, Germany) and cDNA was synthesized using a first-strand cDNA synthesis kit (Roche, Germany). Real-time quantitative RT–PCR was performed using SYBR Green Master (Roche Applied Science, Germany). cDNA was amplified by real-time PCR (LightCycler 480 Instrument II; Roche, Germany) according to the manufacturer’s recommendations. The primers used for amplifying PCR products were: GAT GAT CCT TGA CAG CCT CC (forward primer for *TMEM16A*), GCC ACG GTC TTC TTC TCT GT (reverse primer for *TMEM16A*), TTG AGG TCA ATG AAG GGG TC (forward primer for *GAPDH*), and GAA GGT GAA GGT CGG AGT CA (reverse primer for *GAPDH*). Gene expression was quantified using the comparative threshold cycles of *GAPDH* as the reference gene.

### 2.7. Wound healing assay

FRO cells were plated (5 × 10^5^ cells/mL) in 35-mm μ-Dishes (ibidi, Germany) in 70 μL volume and incubated for 24 h. After cell attachment, the culture insert was gently removed using sterile tweezers. The used well was filled with pre-warmed cell culture medium supplemented with or without the inhibitor, T16Ainh-A01 (2-[(5-ethuy1-1,6-dihydro-4-methy1-6-oxo-2-pyrimidiny1) thio]-N-[4-4(4-methoxypheny1)-2-thiazoyoly1] acetamide; Sigma-Aldrich, St. Louis, MO, USA). Cells were photographed at low magnification (40× amplification) with a real-time cell history recorder (JuLI Stage; NanoEnTeK Inc, MA, USA) at 0 and 18 h. This experiment was repeated thrice.

### 2.8. Cell invasion assay

Cell invasion activity was evaluated in transwell 24-insert plate chambers (8-µm pore size Corning Costar Transwell Permeable Supports; Fisher Scientific, Waltham, MA, USA) coated with BioCoat Matrigel (BD Matrigel Basement Membrane Matrix; diluted 1:50 in RPMI-1640 serum-free medium). After transfection with *ANO1* siRNA or negative control siRNA for 24 h, 3 × 10^5^ FRO cells/mL were plated in upper chambers in 100 μL of serum-free RPMI medium. The lower chambers were filled with RPMI medium supplemented with 10% FBS. The transwells were incubated for 24 h to allow for cell migration. Following incubation, cells from the upper side of the insert filter were completely removed using a cotton swab and cells that invaded through the collagen-coated membrane were fixed in methanol and stained with a Hemacolor rapid staining kit (Merck Millipore, Germany). For quantification, cells were counted under a microscope (Axiocam HRc; Zeiss, Jena, Germany) in six random fields at 20× magnification. These experiments were performed in triplicate.

### 2.9. Statistical analysis

To analyze immunohistochemical data, Pearson’s Chi-squared tests and one-way analysis of variance (ANOVA) were performed with IBM SPSS Software Version 20.0 for Windows (IBM, Armonk, NY, USA). Invasion assay results were analyzed by the Kruskal–Wallis test. Differences with *P* values of less than 0.05 were considered statistically significant.

## 3. Results

### 3.1. Expression profile of ANO1 in human thyroid tumor tissues

**Figure 1.**
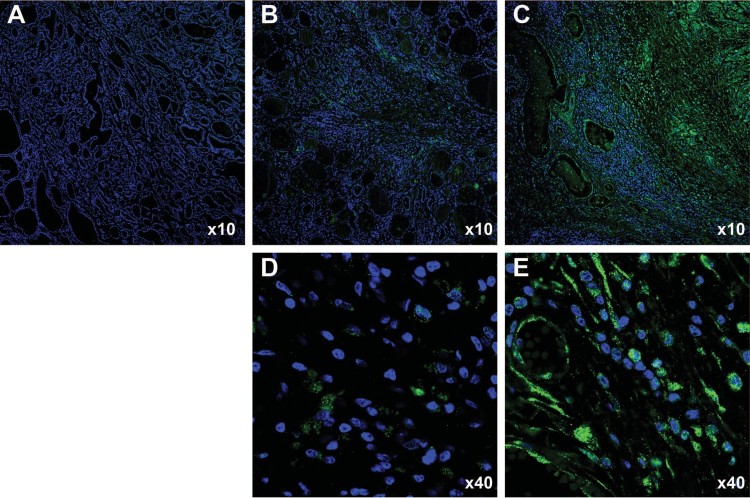
Immunofluorescent staining of thyroid tumor specimens for detecting ANO1. (A) ANO1 expression was not detected in thyroid nodular hyperplasia specimens. (B, D) ANO1 expression was weak in thyroid papillary carcinoma specimens (green dot). (C, E) ANO1 protein was highly expressed in anaplastic thyroid carcinoma (ATC) tissue.

We examined ANO1 expression in patients with benign thyroid tumors, papillary thyroid carcinoma, and ATC (Figure 1). Immunofluorescence staining with an antibody against ANO1 revealed high expression of ANO1 in one ATC sample among 4 different patient-derived samples (Figure 1C and E), while ANO1 expression was scarcely observed in all thyroid nodular hyperplasia and papillary thyroid carcinoma samples (each 20 samples; Figure 1A, B and D). We observed clear ANO1 signal in the cytoplasm (Figure 1E). Furthermore, ANO1-expressing cells in ATC tissue (Figure 1E) showed morphologically extended cell body shape compared to the shape of papillary thyroid carcinoma cells (Figure 1D). According to the 8th American Joint Committee on Cancer (AJCC), the stage of the 3 ATC samples lacking ANO1 expression was stage IVB, while the stage of the ANO1-expressing ATC sample was stage IVC. The stage IVC is a distant metastatic ATC, but the stage IVB has no distant metastasis. Thus, these results suggest that the degree of ANO1 induction may be associated with the aggresivenss of ATC.

### 3.2. Expression of ANO1 was correlated with cell invasion in ATC cells

**Figure 2.**
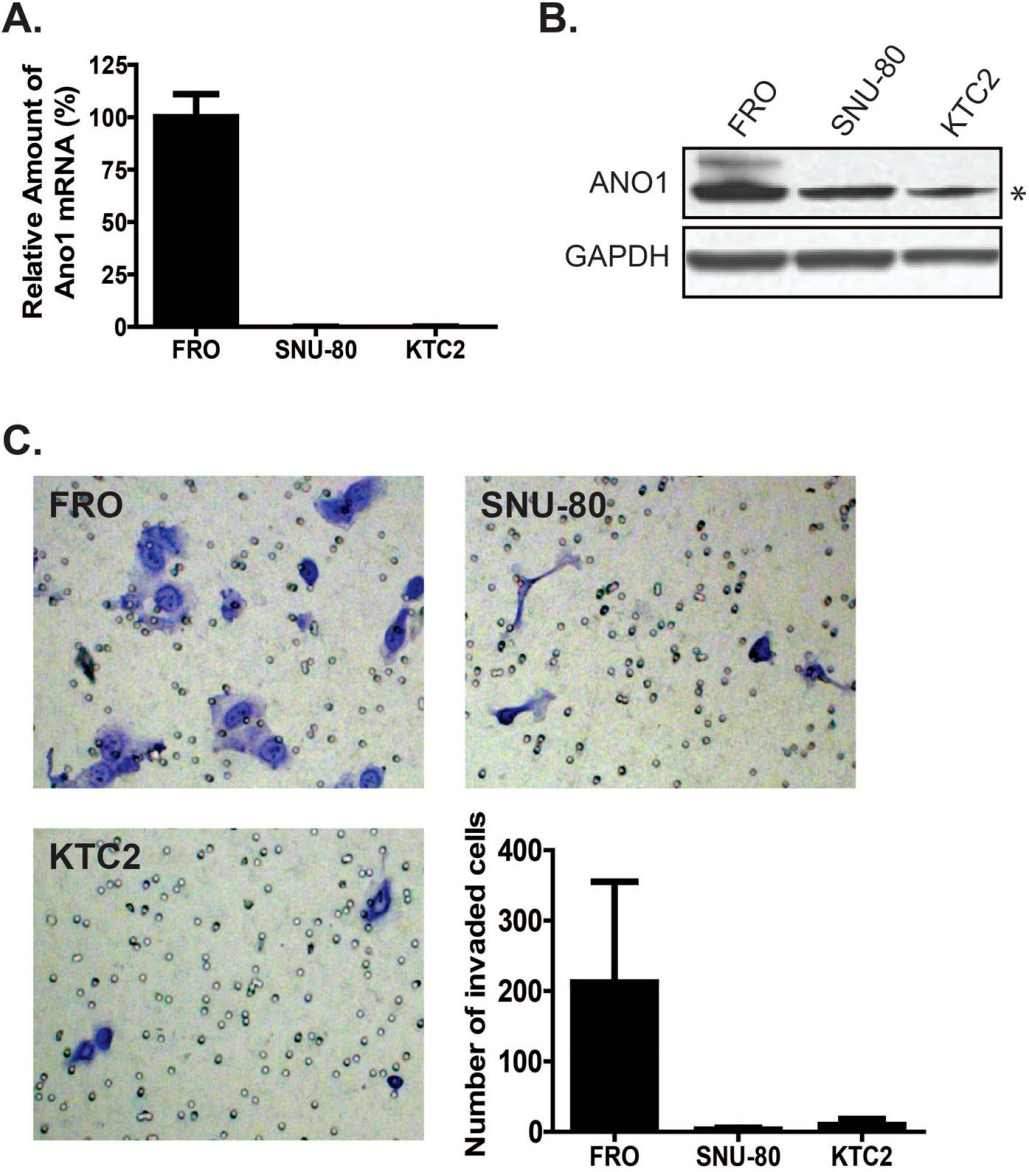
ANO1 expression in anaplastic thyroid carcinoma (ATC) cell lines. (A) qRT-PCR data showed that *ANO1* expression was high in FRO cells. (B) Western blot analysis. ANO1 protein expression was observed in FRO cells (upper band). Lower band (asterisk) was non-specific protein. (C) Cell invasion assay. Stained cells indicate cells that transmigrated across the Matrigel-coated transwell insert. The graph shows the number of migrated cells for each cell line. Among the ATC cell lines examined in this study, the number of migrated cells showed the following trend: FRO > KTC2 > SNU-80.

To identify the potential role of ANO1 in ATC, we first characterized the expression level of *ANO1* in three classic human ATC cell lines: FRO, SNU80, and KTC2 cells (Figure 2). Quantitative analysis of mRNA by real-time RT–PCR showed a significantly higher level of *ANO1* expression in FRO cells as compared to other cell types (Figure 2A). Accordingly, FRO cells exhibited higher ANO1 protein levels as shown Figure 2(B). Next, we performed a Matrigel-coated invasion assay to evaluate the invasive ability of these three cell lines. As shown in Figure 2(C), among the ATC cell lines, FRO cells exhibited the greatest number of invaded cells, and the trend in the tested cells lines followed the order of FRO > KTC2 > SNU-80 (*p* < 0.01). Thus, cell lines with higher levels of *ANO1* exhibited more invasiveness than cell lines with lower *ANO1* expression.

### 3.3. Inhibition of ANO1 activity decreases the migration and invasiveness of ATC cells

**Figure 3.**
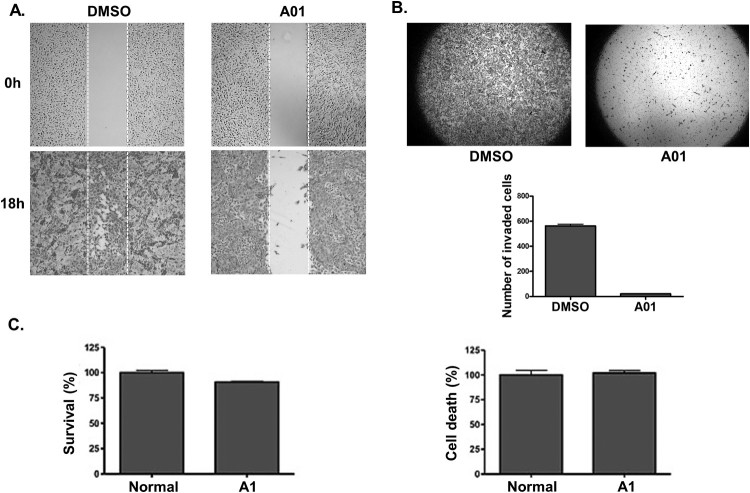
Inhibition of ANO1 activity by T16Ainh-A01 treatment resulted in reduced migration and invasion abilities of FRO cells. (A) Wound healing (or migration) assay in FRO cells treated with DMSO (control) or A01 (10 μM for 18 h). (B) Matrigel-coated transwell invasion assay in FRO cells treated with DMSO (control) or A01 (10 μM for 24 h). (C, D) Effect of T16Ainh-A01 treatment on cell survival (C; CCK-8 assay) and death (D; LDH assay), respectively.

We examined whether the inactivation of ANO1 with a selective CaCC inhibitor, T16Ainh-A01, affected the migration and invasion of FRO cells. T16Ainh-A01 is known to promote proteasome-dependent degradation of ANO1 and was proposed as potential anticancer drug (Bill et al. 2014). We first conducted an invasion assay with Matrigel-coated transwells to evaluate the invasion ability of FRO cells lacking ANO1 expression. T16Ainh-A01-treated FRO cells showed a significant reduction in cell invasiveness as compared with the negative control (dimethyl sulfoxide [DMSO]-treated) cells (Figure 3A). To further determine the effect of T16Ainh-A01 treatment on the migration of FRO cells, we also performed a migration/wound healing assay. As shown Figure 3(B), the wound area closed rapidly (within 18 h) in DMSO-treated cells, but the wound healing process was delayed in T16Ainh-A01-treated cells. On the other hand, survival and cell death of FRO cells were not affected by T16Ainh-A01 treatment, as examined by a CCK-8 survival/growth assay (Figure 3C) and lactate dehydrogenase (LDH) cytotoxicity assay (Figure 3D). Our results showed that T16Ainh-A01 treatment had no effect on FRO cell survival or growth (Figure 3C). Taken together, these results strongly suggest that the suppression of ANO1 activity inhibits the migration and invasion of human thyroid cancer cells.

### 3.4. Silencing ANO1 expression with siRNA transfection decreases the invasion of ATC cells

**Figure 4.**
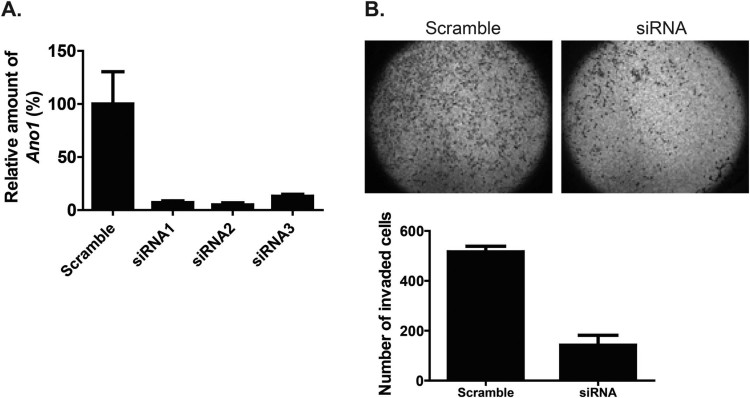
Effect of *ANO1* knockdown using siRNA in FRO cells. (A) Realtime RT-PCR analysis confirmed decreased ANO1 expression in siRNA-transfected FRO cells compared to the expression in the scramble group. (B) Upper images indicate the invaded cells. The lower graph represents the number of invaded cells.

The results presented in Figure 3 suggest that ANO1 activity is required for the migration and invasion of FRO cells. Because pharmacological tests might have off-target effects, we decided to examine the effect of knocking down *ANO1* in FRO cells with three different siRNAs. As shown in Figure 4(A), the expression level of *ANO1* was significantly decreased by all of three siRNAs targeted toward *ANO1*, and we used a mixture of siRNA1 and 2 (siANO1-2). Interestingly, a significant decrease in the number of invading cells was observed in FRO cells transfected with siANO1-2 compared to the number in cells transfected with control siRNA (*p* < 0.05) (Figure 4B). These results support the idea that *ANO1* expression promotes the invasive ability of FRO cells.

## 4. Discussion

In the present study, we observed elevated ANO1 expression only in advanced-stage human ATC tissue. Among the 4 ATC tissues we examined, only the tissue exhibiting the highest malignancy score exhibited enhanced ANO1 expression. Especially, cells that actively expressed ANO1 showed long spindle shape-like metastatic characteristics, also suggesting that ANO1 expression is associated with ATC cell aggressiveness.

*ANO1* gene amplification or mutation is not clear in ATC, but the amplification of chromosomal loci containing the gene encoding ANO1 (11q13 region) is frequently found in various human cancers such as breast, urinary bladder, prostate, and lung cancer; head and neck squamous cell carcinoma (HNSCC); and glioblastoma (Proctor et al. 1991; Liu et al. 2012; Jia et al. 2015). It has been reported that patients with HNSCC malignancies overexpressing ANO1 have worse prognosis (Duvvuri et al. 2012). Our observation of enhanced ANO1 expression in one ATC tissue sample is obviously too preliminary to conclude the importance of ANO1 in ATC. However, combined with evidence from other cancers, we hypothesized that ANO1 plays a significant role in ATC metastasis, and we decided to explore the potential role of ANO1 in ATC *in vitro*.

The potential significance of ANO1 expression on ATC was tested in the ATC cell line FRO which expresses a high level of ANO1. Our results consistently suggest that ANO1 expression is required for the several aspects of cancer, such as increased cell migration and invasion. Malignant cells exhibit key hallmarks such as the ability to invade into surrounding tissues and metastasize (Hanahan and Weinberg 2000). ATC is one of the most aggressive solid tumors that contributes to 14-50% of annual mortality associated with thyroid cancer (Nagaiah et al. 2011). ATC poses a tremendous threat upon its metastasis to vital regions in the neck, ultimately leading to death (Nagaiah et al. 2011). Furthermore, Ruiz et al. has proposed that ANO1 helps regulate cell volume and thereby supports cell migration which facilitates metastasis (Ruiz et al. 2012).

We showed that the effects of ANO1 on migration and invasiveness of FRO cells were simultaneously attenuated by reducing *ANO1* expression as well as inactivating ANO1 activity, respectively. Consistent with the results of our study with ATC cells, T16Ainh-A01 treatment was previously shown to suppress the migration and invasiveness of other cancer types such as glioblastoma, HNSCC, and lung cancer (Jia et al. 2015; Lee et al. 2016). In addition, siRNA-mediated knockdown of *ANO1* decreased cell migration and cell volume in HNSCC cells (Ruiz et al. 2012). In agreement with these previous results, our study provides important evidence that suppressing the function of ANO1 may prevent the migration and invasion of ATC cells. Duvvuri U, et al., reported that ANO1-induced cancer cell proliferation and tumor growth were accompanied by an increase in extracellular signal–regulated kinase (ERK)1/2 activation and cyclin D1 induction (Duvvuri et al. 2012), and inactivation of MEK/ERK and genetic inhibition of ERK1/2 suppressed the growth effect of ANO1, indicating a role for mitogen-activated protein kinase (MAPK) activation in ANO1-mediated proliferation. However, we failed to observe a significant effect of ANO1 suppression on cell survival and cell death in FRO cells (Figure 3), suggesting that the role of ANO1 might depend on the cell type or malignancy stage.

In conclusion, this study proposes that ANO1 expression may serve as an important factor in ATC cell. This finding is not only useful for developing novel pharmacological interventions but also for more specific classification of the severity of thyroid cancer or other types of cancers with upregulated ANO1 expression. Based on these findings, we also suggest the possibility of using ANO1 as a novel biomarker to determine the level of aggressiveness in anaplastic thyroid carcinoma.
